# Methyl 1-benzyl-5-methyl-2,4-diphenyl-1*H*-pyrrole-3-carboxyl­ate

**DOI:** 10.1107/S1600536814003316

**Published:** 2014-02-22

**Authors:** Justin M. Lopchuk, Gordon W. Gribble, Jerry P. Jasinski

**Affiliations:** a Department of Chemistry, Dartmouth College, Hanover, New Hampshire 03755-3564, USA; b Department of Chemistry, Keene State College, 229 Main Street, Keene, NH 03435-2001, USA

## Abstract

In the title compound, C_26_H_23_NO_2_, the dihedral angles between the pyrrole ring and the two phenyl rings are 58.1 (6) and 71.5 (5)°. The mean planes of the 5-methyl­benzene ring and the carboxyl group are twisted by 89.5 (3) and 22.1 (9)°, respectively, from the pyrrole ring. In the crystal, weak C—H⋯O inter­actions lead to supra­molecular layers in the *ab* plane.

## Related literature   

For previous münchnone-based approaches to atorvastatin, see: Pandey & Rao (2004 ▶); Park *et al.* (2008 ▶); Roth *et al.* (1991 ▶). For other examples of the synthesis of pyrroles *via* 1,3-dipolar cyclo­additions with münchnones, see: Lopchuk & Gribble (2011*a* ▶,*b* ▶); Lopchuk *et al.* (2013 ▶). For related crystal structures, see: Grassi *et al.* (2002 ▶); Fang *et al.* (2012 ▶); Donohoe *et al.* (2010 ▶); Sun *et al.* (2004 ▶); Zhang *et al.* (2011 ▶).
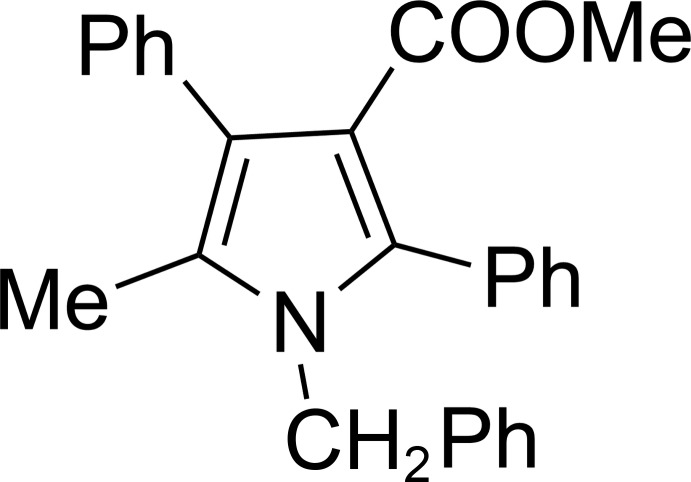


## Experimental   

### 

#### Crystal data  

C_26_H_23_NO_2_
*M* *_r_* = 381.45
Orthorhombic, 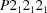

*a* = 8.8056 (2) Å
*b* = 10.6638 (2) Å
*c* = 21.8315 (5) Å
*V* = 2050.00 (8) Å^3^
*Z* = 4
Cu *K*α radiation
μ = 0.61 mm^−1^
*T* = 173 K
0.28 × 0.22 × 0.12 mm

#### Data collection  

Agilent Xcalibur (Eos Gemini) diffractometer
Absorption correction: multi-scan (*CrysAlis PRO* and *CrysAlis RED*; Agilent, 2012 ▶) *T*_min_ = 0.720, *T*_max_ = 1.000
12873 measured reflections
3991 independent reflections
3525 reflections with *I* > 2σ(*I*)
*R*_int_ = 0.041

#### Refinement  

*R*[*F* ^2^ > 2σ(*F* ^2^)] = 0.044
*wR*(*F* ^2^) = 0.119
*S* = 1.06
3991 reflections
264 parameters
H-atom parameters constrained
Δρ_max_ = 0.24 e Å^−3^
Δρ_min_ = −0.20 e Å^−3^
Absolute structure: Flack parameter determined using 1348 quotients (Parsons *et al.*, 2013 ▶)
Absolute structure parameter: 0.02 (18)

### 

Data collection: *CrysAlis PRO* (Agilent, 2012 ▶); cell refinement: *CrysAlis PRO*; data reduction: *CrysAlis RED* (Agilent, 2012 ▶); program(s) used to solve structure: *SUPERFLIP* (Palatinus *et al.*, 2012 ▶); program(s) used to refine structure: *SHELXL97* (Sheldrick, 2008 ▶); molecular graphics: *OLEX2* (Dolomanov *et al.*, 2009 ▶); software used to prepare material for publication: *OLEX2*.

## Supplementary Material

Crystal structure: contains datablock(s) I. DOI: 10.1107/S1600536814003316/tk5295sup1.cif

Structure factors: contains datablock(s) I. DOI: 10.1107/S1600536814003316/tk5295Isup2.hkl

Supporting information file. DOI: 10.1107/S1600536814003316/tk5295Isup3.cml

CCDC reference: 986712

Additional supporting information:  crystallographic information; 3D view; checkCIF report

## Figures and Tables

**Table 1:** Hydrogen-bond geometry (Å, °)

*D*—H⋯*A*	*D*—H	H⋯*A*	*D*⋯*A*	*D*—H⋯*A*
C17—H17*B*⋯O1^i^	0.99	2.56	3.177 (3)	120
C26—H26*A*⋯O2^ii^	0.98	2.59	3.383 (3)	138

Symmetry codes: (i) 
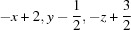
; (ii) 
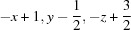
.

**Fig. 1.**
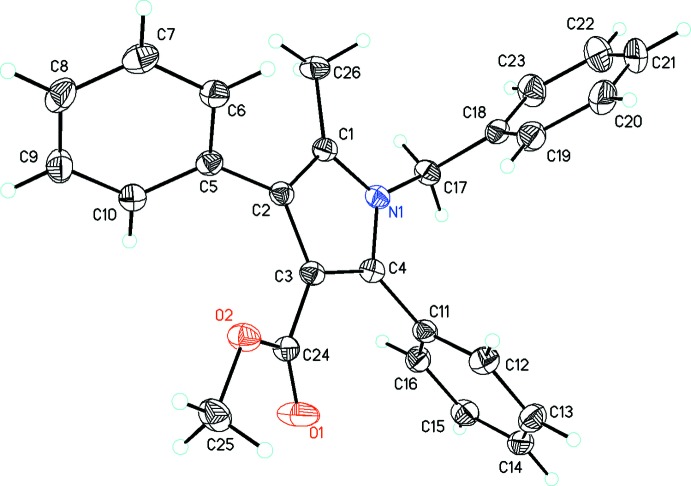
ORTEP drawing of (I), C26H23NO2, showing the labeling scheme with 30% probability displacement ellipsoids.

**Fig. 2.**
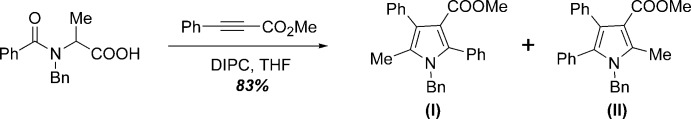
Reaction scheme for C26H23NO2.

## supplementary crystallographic information

### 2. Structural commentary

During the course of our studies toward a total synthesis of atorvastatin, methyl 1-benzyl-5-methyl-2,4-di­phenyl-1*H*-pyrrole-3-carboxyl­ate (I), a penta­substituted pyrrole, was generated from the reaction of a münchnone with methyl 3-phenyl­propiolate. Previously published work on münchnone-based routes toward atorvastatin found that the key münchnone cyclo­additions were either low yielding or unselective and delivered 1:1 mixtures of regioisomers (Pandey *et al.*, 2004; Park *et al.*, 2008; Roth *et al.*, 1991). However, our recent studies on the 1,3-dipolar cyclo­addition of münchnones showed that high regioselectivies can be obtained by proper selection and combination of the münchnone, dipolarophile, and solvent (Lopchuk *et al.*, 2011*a*, Lopchuk *et al.*, 2011*b*; Lopchuk *et al.*, 2013).

A variety of related highly substituted pyrrole crystal structures have been reported including *N*-((2-methyl-4,5-di­phenyl-1*H*-pyrrole-3-carbonyl)­oxy)benzamide (Grassi *et al.*, 2002), *N*,1,5-tri­benzyl-4-(2-chloro­phenyl)-2-methyl-1*H*-pyrrole-3-carbo­thio­amide (Fang *et al.*, 2012), 1-(2-benzyl-5-iso­propyl-4-phenyl-1*H*-pyrrol-3-yl)-2-methyl­propan-1-one (Sun *et al.*, 2004), 5-(4-fluoro­phenyl)-2-iso­propyl-*N*,4-di­phenyl-1*H*-pyrrole-3-carboxamide (Donohoe *et al.*, 2010), and 1-(2-benzoyl-5-iso­propyl-4-phenyl-1*H*-pyrrol-3-yl)-2-methyl­propan-1-one (Zhang *et al.*, 2011). In continuation of our work on münchnone-based routes this paper reports the crystal structure of the title compound, (I), C_26_H_23_NO_2_,

In (I), the dihedral angles between the mean planes of the two phenyl rings (C5–C10 and C11–C16) with that of the pyrrole ring (N1/C1–C4) are 58.1 (6) and 71.5 (5)°, respectively (Fig. 1). The mean planes of the 5-methyl benzene ring (C18–C23) and carboxyl group (O1/O2/C24/C25) are also twisted by 89.5 (3) and 22.1 (9)°, respectively, from that of the pyrrole ring. In the crystal, while no classical hydrogen bonds are observed, weak C—H···O inter­molecular inter­actions are observed (Table 1) which lead to supra­molecular layers in the *ab* plane.

### 5. Synthesis and crystallization

A round bottom flask was charged with *N*-benzoyl-*N*-benzyl­alanine (424 mg, 1.5 mmol), methyl 3-phenyl­propiolate (80 mg, 0.5 mmol), and dry THF (20 ml). The reaction was placed under nitro­gen and *N*,*N*'-diiso­propyl­carbodi­imide (234 ml, 1.5 mmol) added at room temperature. The mixture was heated to reflux for 24 h (Fig. 2). The reaction was cooled to room temperature and concentrated *in vacuo*. The residue was directly purified by flash chromatography to afford pyrrole I as a clear, colorless oil which solidified upon standing (158 mg, 83%). Pyrrole I was obtained as the major isomer (96:4 ratio of I:II). Single crystals suitable for diffraction were grown from di­chloro­methane (slow evaporation) at ambient temperature [M.pt. 454- 455 K].

### 6. Refinement

All of the H atoms were placed in their calculated positions and then refined using the riding model with atom—H lengths of 0.95 Å (CH), 0.99 Å (CH_2_) or 0.98 Å (CH_3_). Isotropic displacement parameters for these atoms were set to 1.2 (CH, CH_2_) or 1.5 (CH_3_) times *U*_eq_ of the parent atom. Idealized methyl groups were refined as rotating.

### Crystal data

**Table:**

C_26_H_23_NO_2_	*D*_x_ = 1.236 Mg m^−^^3^
*M**_r_* = 381.45	Cu *K*α radiation, λ = 1.5418 Å
Orthorhombic, *P*2_1_2_1_2_1_	Cell parameters from 4659 reflections
*a* = 8.8056 (2) Å	θ = 4.1–72.4°
*b* = 10.6638 (2) Å	µ = 0.61 mm^−^^1^
*c* = 21.8315 (5) Å	*T* = 173 K
*V* = 2050.00 (8) Å^3^	Irregular, colourless
*Z* = 4	0.28 × 0.22 × 0.12 mm
*F*(000) = 808	

### Data collection

**Table:**

Agilent Xcalibur (Eos Gemini) diffractometer	3991 independent reflections
Radiation source: Enhance (Cu) X-ray Source	3525 reflections with *I* > 2σ(*I*)
Graphite monochromator	*R*_int_ = 0.041
Detector resolution: 16.0416 pixels mm^-1^	θ_max_ = 72.6°, θ_min_ = 4.1°
ω scans	*h* = −10→10
Absorption correction: multi-scan (*CrysAlis PRO* and *CrysAlis RED*; Agilent, 2012)	*k* = −13→11
*T*_min_ = 0.720, *T*_max_ = 1.000	*l* = −26→26
12873 measured reflections	

### Refinement

**Table:**

Refinement on *F*^2^	Hydrogen site location: inferred from neighbouring sites
Least-squares matrix: full	H-atom parameters constrained
*R*[*F*^2^ > 2σ(*F*^2^)] = 0.044	*w* = 1/[σ^2^(*F*_o_^2^) + (0.0691*P*)^2^] where *P* = (*F*_o_^2^ + 2*F*_c_^2^)/3
*wR*(*F*^2^) = 0.119	(Δ/σ)_max_ < 0.001
*S* = 1.06	Δρ_max_ = 0.24 e Å^−^^3^
3991 reflections	Δρ_min_ = −0.20 e Å^−^^3^
264 parameters	Absolute structure: Flack parameter determined using 1348 quotients [(*I*^+^)-(*I*^-^)]/[(*I*^+^)+(*I*^-^)] (Parsons *et al.*, 2013)
0 restraints	Absolute structure parameter: 0.02 (18)
Primary atom site location: structure-invariant direct methods	

### Special details

**Table:**

Geometry. All e.s.d.'s (except the e.s.d. in the dihedral angle between two l.s. planes) are estimated using the full covariance matrix. The cell e.s.d.'s are taken into account individually in the estimation of e.s.d.'s in distances, angles and torsion angles; correlations between e.s.d.'s in cell parameters are only used when they are defined by crystal symmetry. An approximate (isotropic) treatment of cell e.s.d.'s is used for estimating e.s.d.'s involving l.s. planes.

### Fractional atomic coordinates and isotropic or equivalent isotropic displacement parameters (Å^2^)

**Table:**

	*x*	*y*	*z*	*U*_iso_*/*U*_eq_	
O1	0.9445 (2)	0.4206 (2)	0.84521 (12)	0.0598 (7)	
O2	0.7077 (2)	0.43820 (19)	0.87973 (9)	0.0393 (5)	
N1	0.7361 (2)	0.1689 (2)	0.72021 (10)	0.0315 (5)	
C1	0.6106 (3)	0.1444 (2)	0.75598 (12)	0.0309 (5)	
C2	0.6162 (3)	0.2209 (2)	0.80673 (12)	0.0286 (5)	
C3	0.7518 (3)	0.2943 (2)	0.80125 (11)	0.0288 (5)	
C4	0.8227 (3)	0.2603 (2)	0.74693 (12)	0.0310 (6)	
C5	0.5074 (3)	0.2152 (2)	0.85815 (12)	0.0307 (5)	
C6	0.3528 (3)	0.2348 (3)	0.84848 (13)	0.0366 (6)	
H6	0.3174	0.2554	0.8086	0.044*	
C7	0.2498 (3)	0.2246 (3)	0.89631 (15)	0.0451 (7)	
H7	0.1448	0.2385	0.8890	0.054*	
C8	0.2991 (4)	0.1945 (3)	0.95452 (15)	0.0467 (7)	
H8	0.2284	0.1861	0.9871	0.056*	
C9	0.4528 (4)	0.1765 (3)	0.96491 (14)	0.0458 (7)	
H9	0.4876	0.1573	1.0050	0.055*	
C10	0.5561 (3)	0.1864 (3)	0.91732 (13)	0.0369 (6)	
H10	0.6611	0.1734	0.9250	0.044*	
C11	0.9580 (3)	0.3149 (2)	0.71660 (11)	0.0319 (6)	
C12	0.9505 (3)	0.4354 (3)	0.69197 (14)	0.0402 (6)	
H12	0.8580	0.4811	0.6938	0.048*	
C13	1.0765 (4)	0.4889 (3)	0.66490 (15)	0.0437 (7)	
H13	1.0706	0.5715	0.6488	0.052*	
C14	1.2107 (3)	0.4232 (3)	0.66111 (13)	0.0413 (7)	
H14	1.2972	0.4604	0.6425	0.050*	
C15	1.2193 (3)	0.3033 (3)	0.68443 (14)	0.0419 (7)	
H15	1.3114	0.2573	0.6813	0.050*	
C16	1.0935 (3)	0.2494 (3)	0.71249 (14)	0.0386 (6)	
H16	1.1005	0.1672	0.7290	0.046*	
C17	0.7718 (3)	0.1039 (3)	0.66289 (12)	0.0355 (6)	
H17A	0.7508	0.0134	0.6684	0.043*	
H17B	0.8818	0.1131	0.6546	0.043*	
C18	0.6847 (3)	0.1500 (3)	0.60767 (13)	0.0357 (6)	
C19	0.6183 (4)	0.2672 (3)	0.60486 (15)	0.0450 (7)	
H19	0.6225	0.3213	0.6394	0.054*	
C20	0.5454 (4)	0.3065 (4)	0.55163 (18)	0.0598 (9)	
H20	0.5000	0.3873	0.5500	0.072*	
C21	0.5387 (5)	0.2287 (4)	0.50119 (17)	0.0668 (11)	
H21	0.4888	0.2555	0.4649	0.080*	
C22	0.6049 (5)	0.1122 (4)	0.50404 (16)	0.0688 (11)	
H22	0.6016	0.0585	0.4694	0.083*	
C23	0.6765 (4)	0.0727 (3)	0.55703 (15)	0.0529 (8)	
H23	0.7205	−0.0086	0.5586	0.063*	
C24	0.8137 (3)	0.3888 (2)	0.84308 (12)	0.0316 (5)	
C25	0.7587 (4)	0.5348 (3)	0.92114 (14)	0.0446 (7)	
H25A	0.7974	0.6061	0.8975	0.067*	
H25B	0.8397	0.5016	0.9473	0.067*	
H25C	0.6736	0.5623	0.9467	0.067*	
C26	0.5022 (3)	0.0412 (3)	0.73991 (14)	0.0387 (6)	
H26A	0.4606	0.0560	0.6989	0.058*	
H26B	0.4192	0.0394	0.7698	0.058*	
H26C	0.5558	−0.0393	0.7405	0.058*	

### Atomic displacement parameters (Å^2^)

**Table:**

	*U*^11^	*U*^22^	*U*^33^	*U*^12^	*U*^13^	*U*^23^
O1	0.0366 (12)	0.0751 (16)	0.0676 (15)	−0.0174 (11)	0.0058 (11)	−0.0342 (14)
O2	0.0370 (11)	0.0398 (11)	0.0411 (10)	−0.0007 (8)	0.0007 (8)	−0.0131 (9)
N1	0.0321 (11)	0.0329 (11)	0.0294 (11)	−0.0004 (9)	−0.0020 (8)	−0.0042 (9)
C1	0.0296 (12)	0.0307 (13)	0.0324 (13)	−0.0007 (10)	−0.0031 (10)	−0.0002 (10)
C2	0.0284 (12)	0.0276 (12)	0.0299 (12)	0.0008 (10)	−0.0038 (10)	0.0016 (10)
C3	0.0283 (12)	0.0273 (12)	0.0308 (12)	0.0015 (10)	−0.0030 (10)	−0.0002 (10)
C4	0.0301 (13)	0.0303 (13)	0.0325 (13)	0.0006 (10)	−0.0016 (10)	−0.0015 (10)
C5	0.0318 (13)	0.0263 (12)	0.0339 (13)	−0.0025 (10)	0.0008 (10)	−0.0031 (10)
C6	0.0329 (14)	0.0388 (15)	0.0382 (15)	−0.0016 (11)	−0.0021 (11)	0.0008 (12)
C7	0.0303 (14)	0.0480 (18)	0.0569 (19)	−0.0003 (13)	0.0058 (13)	−0.0027 (14)
C8	0.0483 (17)	0.0467 (17)	0.0452 (17)	−0.0068 (13)	0.0180 (14)	−0.0043 (14)
C9	0.0546 (19)	0.0511 (18)	0.0317 (14)	−0.0035 (15)	0.0047 (13)	0.0018 (13)
C10	0.0349 (14)	0.0403 (15)	0.0354 (14)	−0.0021 (12)	−0.0004 (11)	0.0008 (11)
C11	0.0321 (13)	0.0350 (14)	0.0285 (12)	−0.0026 (10)	0.0000 (10)	−0.0051 (10)
C12	0.0409 (15)	0.0354 (15)	0.0443 (15)	−0.0004 (12)	0.0041 (12)	−0.0057 (13)
C13	0.0517 (18)	0.0359 (16)	0.0436 (16)	−0.0072 (12)	0.0050 (14)	0.0007 (12)
C14	0.0412 (16)	0.0508 (17)	0.0320 (13)	−0.0128 (12)	0.0068 (11)	−0.0053 (13)
C15	0.0316 (15)	0.0529 (17)	0.0413 (15)	0.0013 (12)	0.0022 (11)	−0.0034 (13)
C16	0.0351 (14)	0.0402 (15)	0.0406 (15)	0.0010 (11)	−0.0015 (11)	0.0052 (12)
C17	0.0365 (14)	0.0373 (14)	0.0328 (13)	0.0038 (11)	−0.0005 (11)	−0.0079 (11)
C18	0.0338 (14)	0.0413 (15)	0.0319 (14)	−0.0047 (12)	0.0047 (10)	0.0002 (11)
C19	0.0449 (16)	0.0459 (18)	0.0443 (16)	−0.0021 (14)	0.0027 (13)	0.0018 (13)
C20	0.058 (2)	0.062 (2)	0.060 (2)	−0.0049 (17)	−0.0040 (17)	0.0243 (18)
C21	0.069 (2)	0.094 (3)	0.0374 (17)	−0.016 (2)	−0.0051 (16)	0.0245 (19)
C22	0.086 (3)	0.090 (3)	0.0295 (16)	−0.013 (2)	−0.0009 (17)	−0.0045 (17)
C23	0.064 (2)	0.058 (2)	0.0362 (15)	0.0005 (16)	0.0042 (14)	−0.0082 (15)
C24	0.0309 (13)	0.0313 (13)	0.0325 (13)	−0.0025 (10)	−0.0015 (10)	0.0001 (11)
C25	0.0581 (19)	0.0387 (16)	0.0369 (15)	0.0006 (14)	−0.0044 (14)	−0.0110 (12)
C26	0.0351 (14)	0.0369 (14)	0.0440 (16)	−0.0066 (11)	−0.0027 (12)	−0.0084 (12)

### Geometric parameters (Å, º)

**Table:**

O1—C24	1.202 (3)	C13—H13	0.9500
O2—C24	1.337 (3)	C13—C14	1.376 (4)
O2—C25	1.442 (3)	C14—H14	0.9500
N1—C1	1.379 (3)	C14—C15	1.378 (4)
N1—C4	1.368 (3)	C15—H15	0.9500
N1—C17	1.465 (3)	C15—C16	1.390 (4)
C1—C2	1.377 (4)	C16—H16	0.9500
C1—C26	1.499 (4)	C17—H17A	0.9900
C2—C3	1.433 (3)	C17—H17B	0.9900
C2—C5	1.477 (4)	C17—C18	1.511 (4)
C3—C4	1.388 (4)	C18—C19	1.381 (4)
C3—C24	1.465 (4)	C18—C23	1.381 (4)
C4—C11	1.482 (4)	C19—H19	0.9500
C5—C6	1.394 (4)	C19—C20	1.392 (5)
C5—C10	1.395 (4)	C20—H20	0.9500
C6—H6	0.9500	C20—C21	1.380 (6)
C6—C7	1.387 (4)	C21—H21	0.9500
C7—H7	0.9500	C21—C22	1.374 (6)
C7—C8	1.381 (5)	C22—H22	0.9500
C8—H8	0.9500	C22—C23	1.383 (5)
C8—C9	1.385 (5)	C23—H23	0.9500
C9—H9	0.9500	C25—H25A	0.9800
C9—C10	1.385 (4)	C25—H25B	0.9800
C10—H10	0.9500	C25—H25C	0.9800
C11—C12	1.394 (4)	C26—H26A	0.9800
C11—C16	1.386 (4)	C26—H26B	0.9800
C12—H12	0.9500	C26—H26C	0.9800
C12—C13	1.381 (4)		
			
C24—O2—C25	116.0 (2)	C14—C15—H15	119.9
C1—N1—C17	124.5 (2)	C14—C15—C16	120.2 (3)
C4—N1—C1	109.9 (2)	C16—C15—H15	119.9
C4—N1—C17	125.6 (2)	C11—C16—C15	120.4 (3)
N1—C1—C26	121.2 (2)	C11—C16—H16	119.8
C2—C1—N1	108.3 (2)	C15—C16—H16	119.8
C2—C1—C26	130.3 (3)	N1—C17—H17A	108.6
C1—C2—C3	106.6 (2)	N1—C17—H17B	108.6
C1—C2—C5	124.3 (2)	N1—C17—C18	114.8 (2)
C3—C2—C5	128.8 (2)	H17A—C17—H17B	107.5
C2—C3—C24	129.3 (2)	C18—C17—H17A	108.6
C4—C3—C2	107.7 (2)	C18—C17—H17B	108.6
C4—C3—C24	123.0 (2)	C19—C18—C17	123.0 (3)
N1—C4—C3	107.4 (2)	C23—C18—C17	118.1 (3)
N1—C4—C11	122.5 (2)	C23—C18—C19	118.9 (3)
C3—C4—C11	129.8 (2)	C18—C19—H19	119.9
C6—C5—C2	120.8 (2)	C18—C19—C20	120.3 (3)
C6—C5—C10	118.2 (2)	C20—C19—H19	119.9
C10—C5—C2	120.9 (2)	C19—C20—H20	119.8
C5—C6—H6	119.6	C21—C20—C19	120.3 (4)
C7—C6—C5	120.9 (3)	C21—C20—H20	119.8
C7—C6—H6	119.6	C20—C21—H21	120.4
C6—C7—H7	119.8	C22—C21—C20	119.3 (3)
C8—C7—C6	120.3 (3)	C22—C21—H21	120.4
C8—C7—H7	119.8	C21—C22—H22	119.8
C7—C8—H8	120.3	C21—C22—C23	120.5 (4)
C7—C8—C9	119.4 (3)	C23—C22—H22	119.8
C9—C8—H8	120.3	C18—C23—C22	120.7 (4)
C8—C9—H9	119.7	C18—C23—H23	119.6
C10—C9—C8	120.5 (3)	C22—C23—H23	119.6
C10—C9—H9	119.7	O1—C24—O2	122.4 (3)
C5—C10—H10	119.7	O1—C24—C3	125.0 (3)
C9—C10—C5	120.6 (3)	O2—C24—C3	112.6 (2)
C9—C10—H10	119.7	O2—C25—H25A	109.5
C12—C11—C4	119.8 (2)	O2—C25—H25B	109.5
C16—C11—C4	121.5 (2)	O2—C25—H25C	109.5
C16—C11—C12	118.7 (3)	H25A—C25—H25B	109.5
C11—C12—H12	119.7	H25A—C25—H25C	109.5
C13—C12—C11	120.5 (3)	H25B—C25—H25C	109.5
C13—C12—H12	119.7	C1—C26—H26A	109.5
C12—C13—H13	119.8	C1—C26—H26B	109.5
C14—C13—C12	120.4 (3)	C1—C26—H26C	109.5
C14—C13—H13	119.8	H26A—C26—H26B	109.5
C13—C14—H14	120.1	H26A—C26—H26C	109.5
C13—C14—C15	119.8 (3)	H26B—C26—H26C	109.5
C15—C14—H14	120.1		
			
N1—C1—C2—C3	−0.3 (3)	C5—C2—C3—C24	−4.4 (4)
N1—C1—C2—C5	−175.1 (2)	C5—C6—C7—C8	−0.2 (5)
N1—C4—C11—C12	105.7 (3)	C6—C5—C10—C9	0.5 (4)
N1—C4—C11—C16	−74.9 (4)	C6—C7—C8—C9	1.1 (5)
N1—C17—C18—C19	21.3 (4)	C7—C8—C9—C10	−1.2 (5)
N1—C17—C18—C23	−161.1 (3)	C8—C9—C10—C5	0.4 (5)
C1—N1—C4—C3	0.5 (3)	C10—C5—C6—C7	−0.6 (4)
C1—N1—C4—C11	−174.4 (2)	C11—C12—C13—C14	0.9 (5)
C1—N1—C17—C18	80.0 (3)	C12—C11—C16—C15	0.1 (4)
C1—C2—C3—C4	0.6 (3)	C12—C13—C14—C15	0.0 (5)
C1—C2—C3—C24	−179.0 (3)	C13—C14—C15—C16	−0.9 (4)
C1—C2—C5—C6	−59.7 (4)	C14—C15—C16—C11	0.8 (5)
C1—C2—C5—C10	118.3 (3)	C16—C11—C12—C13	−1.0 (4)
C2—C3—C4—N1	−0.7 (3)	C17—N1—C1—C2	178.6 (2)
C2—C3—C4—C11	173.8 (3)	C17—N1—C1—C26	3.4 (4)
C2—C3—C24—O1	157.7 (3)	C17—N1—C4—C3	−178.2 (2)
C2—C3—C24—O2	−22.4 (4)	C17—N1—C4—C11	6.9 (4)
C2—C5—C6—C7	177.4 (3)	C17—C18—C19—C20	177.1 (3)
C2—C5—C10—C9	−177.5 (3)	C17—C18—C23—C22	−176.7 (3)
C3—C2—C5—C6	126.6 (3)	C18—C19—C20—C21	0.0 (5)
C3—C2—C5—C10	−55.4 (4)	C19—C18—C23—C22	0.9 (5)
C3—C4—C11—C12	−67.9 (4)	C19—C20—C21—C22	−0.1 (6)
C3—C4—C11—C16	111.5 (3)	C20—C21—C22—C23	0.6 (6)
C4—N1—C1—C2	−0.1 (3)	C21—C22—C23—C18	−1.0 (6)
C4—N1—C1—C26	−175.3 (2)	C23—C18—C19—C20	−0.4 (5)
C4—N1—C17—C18	−101.5 (3)	C24—C3—C4—N1	178.9 (2)
C4—C3—C24—O1	−21.8 (4)	C24—C3—C4—C11	−6.7 (4)
C4—C3—C24—O2	158.1 (2)	C25—O2—C24—O1	1.6 (4)
C4—C11—C12—C13	178.4 (3)	C25—O2—C24—C3	−178.3 (2)
C4—C11—C16—C15	−179.3 (3)	C26—C1—C2—C3	174.3 (3)
C5—C2—C3—C4	175.1 (2)	C26—C1—C2—C5	−0.6 (4)

### Hydrogen-bond geometry (Å, º)

**Table:**

*D*—H···*A*	*D*—H	H···*A*	*D*···*A*	*D*—H···*A*
C17—H17*B*···O1^i^	0.99	2.56	3.177 (3)	120
C26—H26*A*···O2^ii^	0.98	2.59	3.383 (3)	138

Symmetry codes: (i) −*x*+2, *y*−1/2, −*z*+3/2; (ii) −*x*+1, *y*−1/2, −*z*+3/2.
